# Correlation between gestational diabetes mellitus and postpartum cardiovascular metabolic indicators and inflammatory factors: a cohort study of Chinese population

**DOI:** 10.3389/fendo.2024.1401679

**Published:** 2024-11-25

**Authors:** Xin Zhao, Dan Zhao, Jianbin Sun, Ning Yuan, Xiaomei Zhang

**Affiliations:** Department of Endocrinology, Peking University International Hospital, Beijing, China

**Keywords:** gestational diabetes mellitus, cardiovascular disease, glucose and lipid metabolism, inflammatory factors, cohort study

## Abstract

**Objective:**

This study aimed to analyze the correlation between gestational diabetes mellitus and postpartum metabolic indicators and inflammatory factors, and explore the role of inflammatory factors, so as to provide evidence for the early prevention of postpartum CVD risk in gestational diabetes mellitus.

**Methods:**

This prospective study was based on the pregnant women cohort study established in Peking University International Hospital from December 2017 to March 2019. A total of 120 women were enrolled sequentially, including 60 cases of gestational diabetes mellitus (GDM group) and 60 cases of non-gestational diabetes mellitus (non-GDM group) after 4-7 years. The general information, inflammatory factors and metabolic indicators of the women were collected and analyzed.

**Results:**

(1)The TyG and siMS levels in the GDM group were higher than those in the non-GDM group (p<0.05, respectively). The interleukin-6(IL-6) levels in the GDM group were higher than those in the non-GDM group and the difference was statistically significant (p<0.05). (2) The results of linear regression analysis showed that GDM was associated with postpartum GLU_0min_ (β=0.94, 95%CI: 0.27-1.60, p<0.05), GLU_120min_ (β=2.76, 95%CI: 1.57-3.94, p<0.05) and HbA1c (β=0.49, 95%CI: 0.27-1.60, p<0.05). At the same time, GDM was significantly correlated with postpartum metabolic indicators triglyceride-glucose (TyG) index (β=0.31, 95%CI: 0.01-0.61, p<0.05) and siMS score (β=0.45, 95%CI: 0.03-0.88, p<0.05).The results of linear regression analysis showed that GDM was significantly correlated with IL-6 (β=0.91, 95%CI: 0.02-1.79, p<0.05). (3) Logistic regression analysis showed that GDM was an independent risk factor for postpartum abnormal metabolism (OR=10.62, 95%CI: 1.66-68.17, p<0.05), and an independent risk factor for postpartum high low-density lipoprotein cholesterolemia (OR=3.38, 95%CI: 1.01-11.56, p<0.05). (4) The IL-6 had a mediating effect in the association between GDM and postpartum TyG and siMS, with the mediating effect sizes being 20.59% and 30.77%, respectively.

**Conclusion:**

This study revealed that GDM history can lead to abnormal glucose and lipid metabolism indexes in postpartum women, affect the levels of postpartum CVD-related metabolic indicators. Meanwhile, IL-6 shows a mediating role, providing important clinical evidence for the prevention and control of CVD in such high-risk populations and the improvement of cardiovascular health.

## 1 Introduction

Gestational diabetes mellitus (GDM) is a common metabolic disease during pregnancy, and its adverse effects are not limited to macrosomia, premature delivery, preeclampsia and other adverse pregnancy outcomes (1). The risk of cardiovascular disease (CVD) in GDM women after delivery is also significantly increased (2). A large prospective cohort study of nearly 90,000 people in the United States reported (3) that GDM was associated with increased long-term CVD risk. After adjusting for confounding factors such as body mass index (BMI), diet, physical activity, smoking and other lifestyle factors, the subsequent CVD risk difference between non-GDM and GDM women was still significant (HR=1.29, 95%CI: 1.01-1.65). GDM is a high-risk population for future cardiovascular diseases. Elucidating the possible biological mechanism of GDM postpartum CVD risk is of great significance for early prevention and control of CVD and improving cardiovascular health. The metabolic indicators are powerful predictors of CVD. The levels of fasting blood glucose (GLU_0min_), glycosylated hemoglobin (HbA1c), triglyceride (TG), total cholesterol (TC), high-density lipoprotein cholesterol (HDL-C) and other metabolic indicators are significantly associated with increased CVD risk (4–7). In addition, some comprehensive indicators such as triglyceride-glucose (TyG) and siMS score are also used to predict the occurrence of CVD (8, 9). Currently, studies have reported that GDM is associated with postpartum metabolic indicators. A meta-analysis of GDM and postpartum cardiovascular risk factors showed that compared with women without GDM in the past, the levels of total cholesterol, triglycerides and fasting blood glucose in women with GDM increased significantly as early as 1 year after delivery (10); another Canadian study also found that the levels of blood glucose and blood lipids in women with GDM were in a long-term poor state, leading to an increased risk of CVD (11). However, most of the relevant studies have been carried out in European and American populations (10), and there is a lack of correlation studies between GDM and postpartum metabolic indicators in Chinese population. However, the genetic background and lifestyle that are quite different between Chinese and Western populations are important factors affecting the occurrence of CVD diseases and metabolic indicators (12, 13), which may lead to the fact that these results may not be suitable for direct application in Chinese population. Therefore, it is urgent to carry out correlation studies between GDM and postpartum CVD related metabolic indicators in Chinese population, so as to provide scientific basis for the prevention and control of early postpartum CVD risk in Chinese population. Inflammatory factors play an important role in the occurrence and development of GDM and CVD.

The occurrence and development of CVD are closely related to the role of inflammatory factors. A number of clinical studies and animal experiments have shown that inflammatory factors are involved in the changes of various signaling pathways, and can affect the occurrence and development of CVD by promoting the proliferation and migration of vascular smooth muscle cells and affecting the function of vascular endothelium (14). The main cause of GDM is insulin resistance during pregnancy. Inflammatory response during pregnancy activates the production of various proinflammatory factors by participating in various transcription-mediated molecular pathways, oxidation and metabolic stress. Proinflammatory factors cause insulin resistance by interfering with insulin signaling pathways, thereby increasing the risk of GDM (15, 16). At present, the pathogenic mechanism of the doubling of the risk of postpartum CVD in GDM women is not clear, but since GDM is closely related to the level of inflammation during pregnancy, and inflammatory factors are associated with the increased risk of CVD, given the important role of inflammatory factors in the occurrence and development of both diseases, the increased risk of postpartum CVD in GDM women may be mediated or accelerated by inflammatory factors. However, no relevant population studies have clarified the role of inflammatory factors in the association between the history of GDM and postpartum cardiovascular health.

Therefore, this study aims to explore the association between GDM and postpartum CVD related metabolic indicators, explore the biological mechanism of increased postpartum CVD risk in GDM women, and at the same time, explore the role of inflammatory factors in the association between GDM and postpartum CVD metabolic indicators, comprehensively reveal the influence of GDM on postpartum CVD related metabolic indicators and the possible mechanism, so as to provide important clinical basis for the prevention and control of CVD in such high-risk populations and the improvement of cardiovascular health.

## 2 Materials and methods

### 2.1 Subjects

This prospective study was based on the pregnant women cohort study established in Peking University International Hospital from December 2017 to March 2019. 120 women with gestational diabetes mellitus (GDM group) and 60 women without gestational diabetes mellitus (non-GDM group) were enrolled sequentially during postpartum 4-7 years. The study was approved by the Bioethics Committee of Peking University International Hospital. All protocols followed the ethical guidelines of the institution and national committee and complied with the 1964 Declaration of Helsinki and subsequent amendments. All participants provided written informed consent. The ethics approval number is 2022-KY-0071-01.

Inclusion criteria: (1) Over 18 years old. (2) Willing to enter the cohort, accept the relevant questionnaire survey and agree to collect blood samples after being informed of the relevant investigation content.

Exclusion criteria: (1) Diabetes mellitus (including type 1 diabetes mellitus and type 2 diabetes mellitus) diagnosed before pregnancy; (2) Twin or multiple pregnancy; (3) Rheumatic immune system diseases; (4) Severe liver and kidney insufficiency; (5) Long-term use of antidepressants or corticosteroids.

### 2.2 Research methods

#### 2.2.1 General information and basic information

1. Baseline information: The age of pregnancy, parity history and the results of oral glucose tolerance test (OGTT) in the pregnancy were recorded at the time of enrollment.
2. Postpartum follow-up information: All subjects were followed up 4-7 years after delivery, and blood pressure including systolic blood pressure (SBP) and diastolic blood pressure (DBP) were measured after delivery; meanwhile, height, weight, body fat rate (BFR), waist and hip were measured, and body mass index (BMI) and waist to hip ratio (WHR) were calculated and recorded. BMI was calculated using the formula BMI (kg/m^2^) = weight (kg)/body height^2^(m^2^).

#### 2.2.2 GDM diagnostic criteria

Pregnant women were screened for GDM by 75g OGTT at 24-28 weeks of gestation. Pregnant women were admitted to the hospital in the morning after 8-12 hours of fasting, and given 75g glucose powder dissolved in 250ml-300ml warm boiling water, which was rapidly taken orally within 5 minutes. Venous blood was collected before taking glucose, 1 hour after taking glucose, and 2 hours after taking glucose, respectively, for the detection of blood glucose level.

IADPSG was used as the diagnostic criteria for GDM (17), 1 hour after taking glucose, and 2 hours after taking glucose. The three blood glucose values including fasting blood glucose,1 hour after taking glucose and 2 hours after taking glucose should be lower than 5.1mmol/L, 10.0mmol/L, and 8.5mmol/L, respectively. Any blood glucose value reaching or exceeding the above criteria was diagnosed as GDM.

#### 2.2.3 Detection of postpartum metabolic indicators

During the postpartum follow-up, fasting venous blood was collected by professional medical staff in the clinical department to detect a number of biochemical metabolic indicators, including C-reactive protein (CRP), HbA1c, TC, TG, HDL-C, low-density lipoprotein cholesterol (LDL-C), small and dense low-density lipoprotein cholesterol (SDLDL-C), apolipoprotein A (ApoA), apolipoprotein B (ApoB), lipoprotein A (LPA), free fatty acids (FFA), adiponectin (ADPN) and leptin (LP). TC/HDL-C value was calculated. The HbA1c levels were measured by high-performance liquid chromatography (HPLC) with a Dongcao G8 analyzer.

At the same time, all subjects were given OGTT examination during postpartum follow-up, the method is as follows: all subjects were admitted to hospital in the morning after fasting for 8-12 hours, and 75g glucose powder was dissolved in 250ml-300ml warm boiling water, which was rapidly taken orally within 5 minutes. Venous blood was collected before taking sugar water and 2 hours after taking sugar water, respectively, to detect 0 min blood glucose (GLU_0min)_ and 2 hours blood glucose (GLU_120min_).

According to the values of related metabolic indicators, they were classified: (1) Type 2 diabetes mellitus (T2DM) (18): typical symptoms of diabetes and random blood glucose 11.1 mmol/L; fasting blood glucose≥7.0 mmol/L; in the OGTT, blood glucose≥11.1 mmol/L after taking 75 g glucose for 2 hours. If there were no symptoms of diabetes, the examination was repeated on another day. (2) Impaired glucose regulation (IGR): including impaired fasting blood glucose and impaired glucose tolerance, impaired fasting blood glucose (IFG) refers to fasting (blood glucose 6.1-7.0mmol/L, and OGTT2h blood glucose <7.8mmol/L; impaired glucose tolerance (IGT) refers to fasting blood glucose <6.1mmol/L, and OGTT2h blood glucose 7.8-11.1mmol/L; (3) Abnormal HbA1c: HbA1c≥6.5%; (4) Dyslipidemia: divided into 4 types: ① hypercholesterolemia: TC≥5.2 mmol/L; ② hypertriglyceridemia: TG≥1.7 mmol/L; ③ low high-density lipoprotein cholesterolemia: HDL-C ≤ 1.3 mmol/L; ④ high low-density lipoprotein cholesterolemia: LDL-C≥3.4 mmol/L.

#### 2.2.4 Inflammatory factor detection

The blood samples were centrifuged at 3000 r/min for 6 min. The plasma and blood cells were separated into two 1.5 ml centrifuge tubes with a pipette. The blood was centrifuged and separated within 2 hours after collection, and then placed into a cryogenic box according to the serial number, and returned to the laboratory -80°C refrigerator for preservation. ELISA was used to detect 7 inflammatory factors in the serum of the study subjects, including tumor necrosis factor-α (TNF-α), tumor necrosis factor-β (TNF-β), interleukin-1β (IL-1β), interleukin-6 (IL-6), interleukin-8 (IL-8), interleukin -2 (IL-2) and growth differentiation factor15 (GDP15). The instrument used in this study was MK3 ELISA kit (Thermo, America), and the kit was Thermo’s high-sensitivity human serum factor kit.

#### 2.2.5 Calculation of metabolic indexes

1. Triglyceride-glucose (TyG) index: The TyG index was calculated using the following formula:
$$\text{TyG}=\ln\left(\frac{TG\times Glu0min}{2}\right).$$
2. siMS score: The siMS score was calculated from postpartum waist circumference, height, GLU_0min_, TG, SBP, and HDL-C (19), the siMS score was calculated using the following formula:
$$\text{siMS score}=\;\frac{2\times waist}{height}+\frac{GLU0min}{5.6}+\frac{TG}{1.7}+\frac{SBP}{130}+\frac{HDL-C}{1.28}.$$

### 2.3 Statistical analysis

All data were analyzed using SPSS 22.0. Data were tested for normality, and normally distributed data were expressed as means ± standard deviation (x ± s) and compared using t-tests. Non-normally distributed data were expressed as medians (P25, P75) and compared using rank sum tests. The counting data were expressed as a rate and compared between the two groups using the χ2 test. Binary logistic regression was used to analyze the association between GDM prevalence and postpartum metabolic indicators, linear regression was used to analyze the association between GDM prevalence and postpartum metabolic indicators, and the association between GDM prevalence and inflammatory factors, and PROCESS 3.3 plug-in was used to analyze the mediating role of inflammatory factors in GDM and some of its related postpartum metabolic indicators. All statistical tests were two-sided, and statistical significance was set at p<0.05.

## 3 Results

### 3.1 Comparison of general conditions and biochemical indexes between the two groups

Compared with the non-GDM group, the women in the GDM group were older, with statistically significant differences (p<0.05). Compared with the non-GDM group, the women in the GDM group had significantly higher GLU_0min_, GLU_120min_ and HbA1c levels, with statistically significant differences (p<0.05, respectively). Compared with the non-GDM group, the women in the GDM group had significantly higher SBP and DBP, with statistically significant differences (p<0.05, respectively). Compared with the non-GDM group, the women in the GDM group had significantly higher TC,LDL-C,SDLDL-C,APO-b and FFA, with statistically significant differences (p<0.05, respectively). The TyG and siMS levels in the GDM group were higher than those in the non-GDM group (p<0.05, respectively). The IL-6 levels in the GDM group were higher than those in the non-GDM group and the difference was statistically significant(p<0.05), while there was not significant differences in other inflammatory factors between the two groups(p>0.05, respectively). There was no significant differences in BMI, BFR, WHR between the two groups (p>0.05). Also, there were no significant differences in TG, HDL-C, APOA, LPA, ADPN and LP between the two groups (p>0.05). (Shown as **Table 1**).

**Table 1:** Comparison of general conditions and biochemical indexes between the two groups.

Index	non-GDM group	GDM group	t (X^2^)	P	Index	non-GDM group	GDM group	t (X^2^)	P
	(n=60)	(n=60)				(n=60)	(n=60)		
Age (years)	31.48 ± 3.31	33.03 ± 3.54	-4.13	<0.05	HbA1c (%)	5.45 ± 0.28	5.89 ± 0.93	-3.52	<0.05
BMI (kg/m^2^)	22.99 ± 3.50	23.58 ± 3.91	-0.86	0.39	GLU_0min_ (mmol/L)	5.15 ± 1.15	5.92 ± 1.79	-2.82	<0.05
BFR	32.08 ± 5.78	32.42 ± 6.41	-0.31	0.76	GLU_120min_ (mmol/L)	5.92 ± 1.32	8.52 ± 3.42	-5.49	<0.05
WHR	0.84 ± 0.06	0.83 ± 0.06	0.83	0.41	TyG	8.21 ± 0.59	8.55 ± 0.79	-2.67	<0.05
Follow-up time (years)	5.72 ± 0.98	5.70 ± 1.00	-0.07	0.95	TC/HDL-C	3.44 ± 0.91	3.80 ± 1.13	-1.92	0.06
Parity					siMS	2.31 ± 0.66	2.70 ± 1.25	-2.12	<0.05
0	36 (60%)	30 (50%)	1.21	0.27	CRP (mg/L)	1.23 ± 1.93	1.92 ± 2.28	-1.45	0.15
≥1	24 (40%)	30 (50%)			IL-6 (pg/ml)	2.49 ± 1.68	3.33 ± 2.15	-2.39	<0.05
SBP (mmHg)	109.03 ± 10.54	114.12 ± 15.07	-2.14	<0.05	IL-8 (pg/ml)	7.54 ± 3.26	7.59 ± 3.13	-0.07	0.94
DBP (mmHg)	67.37 ± 7.45	73.02 ± 10.73	-3.35	<0.05	IL-2 (pg/ml)	5.61 ± 3.51	6.03 ± 3.14	-0.68	0.50
TC (mmol/L)	4.50 ± 0.83	4.95 ± 0.78	-3.05	<0.05	IL-1β (pg/ml)	4.88 ± 2.25	4.39 ± 2.26	1.20	0.23
TG (mmol/L)	1.08 ± 0.76	1.45 ± 1.33	-1.86	0.07	TNF-a (pg/ml)	9.16 ± 5.50	9.51 ± 5.10	-0.36	0.72
LDL-C (mmol/L)	2.73 ± 0.70	3.07 ± 0.69	-2.66	<0.05	TNF-β (pg/ml)	16.67 ± 8.51	19.15 ± 6.83	-1.76	0.08
HDL-C (mmol/L)	1.36 ± 0.29	1.37 ± 0.30	-0.18	0.86	GDF15 (pg/ml)	610.62 ± 199.37	588.25 ± 192.78	0.62	0.53
SDLDL-C (mmol/L)	0.82 ± 0.30	0.99 ± 0.45	-2.28	<0.05	LPA (mg/l)	105.97 ± 107.58	144.32 ± 154.14	-1.57	0.12
APO-a (mg/dl)	141.23 ± 22.61	150.93 ± 64.72	-0.36	0.28	FFA (uEq/l)	417.98 ± 228.56	537.44 ± 247.21	-2.72	<0.05
APO-b (mg/dl)	92.05 ± 21.34	104.18 ± 25.05	-1.76	<0.05	ADPN (ng/ml)	2715.72 ± 1005.91	2641.38 ± 1085.36	0.39	0.70
					LP (pg/ml)	7341.04 ± 2494.09	8056.67 ± 2781.86	-1.48	0.14

### 3.2 Correlation between GDM and postpartum metabolic conditions

The results of linear regression analysis showed that after adjusting for age, parity and follow-up time, GDM was significantly correlated with postpartum GLU_0min_ (β=0.94, 95%CI: 0.27-1.60, p<0.05), GLU_120min_ (β=2.76, 95%CI: 1.57-3.94, p<0.05), HbA1c (β=0.49, 95%CI: 0.18-0.79, p<0.05), DBP (β=4.17, 95%CI: 0.17-8.18, p<0.05), TC (β=0.48, 95%CI: 0.12-0.84, p<0.05), LDL-C (β=0.38, 95%CI: 0.08-0.69, p<0.05), SDLDL-C (β=0.19, 95%CI: 0.02-0.37, p<0.05), APO-b (β=13.59, 95%CI: 3.21-23.97, p<0.05), LPA (β=58.61, 95%CI: 3.97-113.25, p<0.05) and FFA (β=132.00, 95%CI: 29.77-234.23, p<0.05).At the same time, after adjusting for age, parity and follow-up time, GDM was significantly correlated with TyG (β=0.31, 95%CI: 0.01-0.61, p<0.05) and siMS (β=0.45, 95%CI: 0.03-0.88, p<0.05).The results of linear regression analysis showed that after adjusting for age, parity and follow-up time, GDM was significantly correlated with IL-6 (β=0.91, 95%CI: 0.02-1.79, p<0.05), but not with other inflammatory factors. (Shown as **Table 2**).

**Table 2:** Correlation between GDM and postpartum metabolic conditions.

Index	Mode 1		Model 2		Index	Mode 1		Model 2	
	βst (95%CI)	p	βst (95%CI)	p		βst (95%CI)	p	βst (95%CI)	p
BMI (kg/m^2^)	0.58 (-0.75,1.91)	0.39	0.58 (-0.99,2.15)	0.47	LP (pg/ml)	715.62 (-229.77,1661.01)	0.14	975.52 (-185.74,2136.78)	0.10
BFR	0.35 (-1.84,2.53)	0.76	0.35 (-2.27,2.98)	0.79	HbA1c (%)	0.44 (0.20,0.69)	<0.05	0.49 (0.18,0.79)	<0.05
WHR	-0.01 (-0.03,0.01)	0.41	-0.02 (-0.04,0.01)	0.21	GLU_0min_ (mmol/L)	0.77 (0.24,1.31)	<0.05	0.94 (0.27,1.60)	<0.05
SBP (mmHg)	5.08 (0.43,9.74)	<0.05	3.85 (-1.69,9.39)	0.18	GLU_120min_ (mmol/L)	2.60 (1.67,3.53)	<0.05	2.76 (1.57,3.94)	<0.05
DBP (mmHg)	5.65 (2.34,8.96)	<0.05	4.17 (0.17,8.18)	<0.05	TyG	0.34 (0.09,0.59)	<0.05	0.31 (0.01,0.61)	<0.05
TC (mmol/L)	0.45 (0.16,0.74)	<0.05	0.48 (0.12,0.84)	<0.05	TC/HDL-C	0.36 (-0.01,0.73)	0.06	0.41 (-0.04,0.85)	0.08
TG (mmol/L)	0.37 (-0.02,0.75)	0.07	0.42 (-0.04,0.89)	0.08	siMS	0.39 (0.03,0.75)	<0.05	0.45 (0.03,0.88)	<0.05
LDL-C (mmol/L)	0.34 (0.09,0.59)	<0.05	0.38 (0.08,0.69)	<0.05	CRP (mg/L)	0.69 (-0.24,1.62)	0.15	1.06 (-0.13,2.24)	0.08
HDL-C (mmol/L)	0.01 (-0.10,0.12)	0.86	0.01 (-0.13,0.12)	0.92	IL-6 (pg/ml)	0.84 (0.15,1.53)	<0.05	0.91 (0.02,1.79)	<0.05
SDLDL-C (mmol/L)	0.16 (0.02,0.30)	<0.05	0.19 (0.02,0.37)	<0.05	IL-8 (pg/ml)	0.04 (-1.10,1.19)	0.094	0.39 (-0.98,1.76)	0.58
APO-a (mg/dl)	9.70 (-7.70,27.09)	0.28	17.88 (-3.46,39.22)	0.10	IL-2 (pg/ml)	0.41 (-0.78,1.60)	0.50	-0.08 (-1.56,1.40)	0.92
APO-b (mg/dl)	12.13 (3.71,20.54)	<0.05	13.59 (3.21,23.97)	<0.05	IL-1β (pg/ml)	-0.49 (-1.30,0.31)	0.23	-0.81 (-1.77,0.15)	0.10
LPA (mg/l)	38.35 (-9.62,86.32)	0.12	58.61 (3.97,113.25)	<0.05	TNF-a (pg/ml)	0.35 (-1.55,2.25)	0.72	-0.44 (2.73,1.86)	0.71
FFA (uEq/l)	119.46 (33.24,205.67)	<0.05	132.00 (29.77,234.23)	<0.05	TNF-β (pg/ml)	2.48 (-0.28,5.24)	0.08	3.26 (-0.12,6.65)	0.06
ADPN (ng/ml)	-74.33 (-448.78,300.11)	0.70	67.22 (-390.26,524.69)	0.77	GDF15 (pg/ml)	-22.37 (-92.55,47.80)	0.53	-0.69 (-86.09,84.63)	0.99

Model 1 did not adjust for other factors, Model 2 adjusted for gestational age, parity and follow-up time.

### 3.3 Correlation between GDM and postpartum metabolic abnormalities

Compared with non-GDM group, GDM group had higher proportion of patients with T2DM and IGR, patients with elevated HbAlc level, patients with hypercholesterolemia and patients with high LDL cholesterolemia, with statistically significant differences (p<0.05, respectively). (shown as **Table 3**).

**Table 3:** Comparison of postpartum metabolic abnormalities between the two groups.

Index	non-GDM group	GDM group	(X^2^)	P
	(n=60)	(n=60)		
T2DM (%)	0 (0%)	12 (20%)	13.33	<0.05
IGR (%)	2 (3.33%)	11 (18.33%)	6.99	<0.05
Abnormal HbA1c (%)	2 (3.33%)	11 (18.33%)	6.99	<0.05
Hypercholesterolemia (%)	11 (18.33%)	21 (35%)	4.26	<0.05
Hypertriglyceridemia (%)	7 (11.67%)	12 (20%)	1.56	0.21
High LDL cholesterolemia (%)	7 (11.67%)	16 (26.67%)	4.36	<0.05
Low HDL cholesterolemia (%)	10 (16.67%)	9 (15%)	0.06	0.80

**Figure 1:**
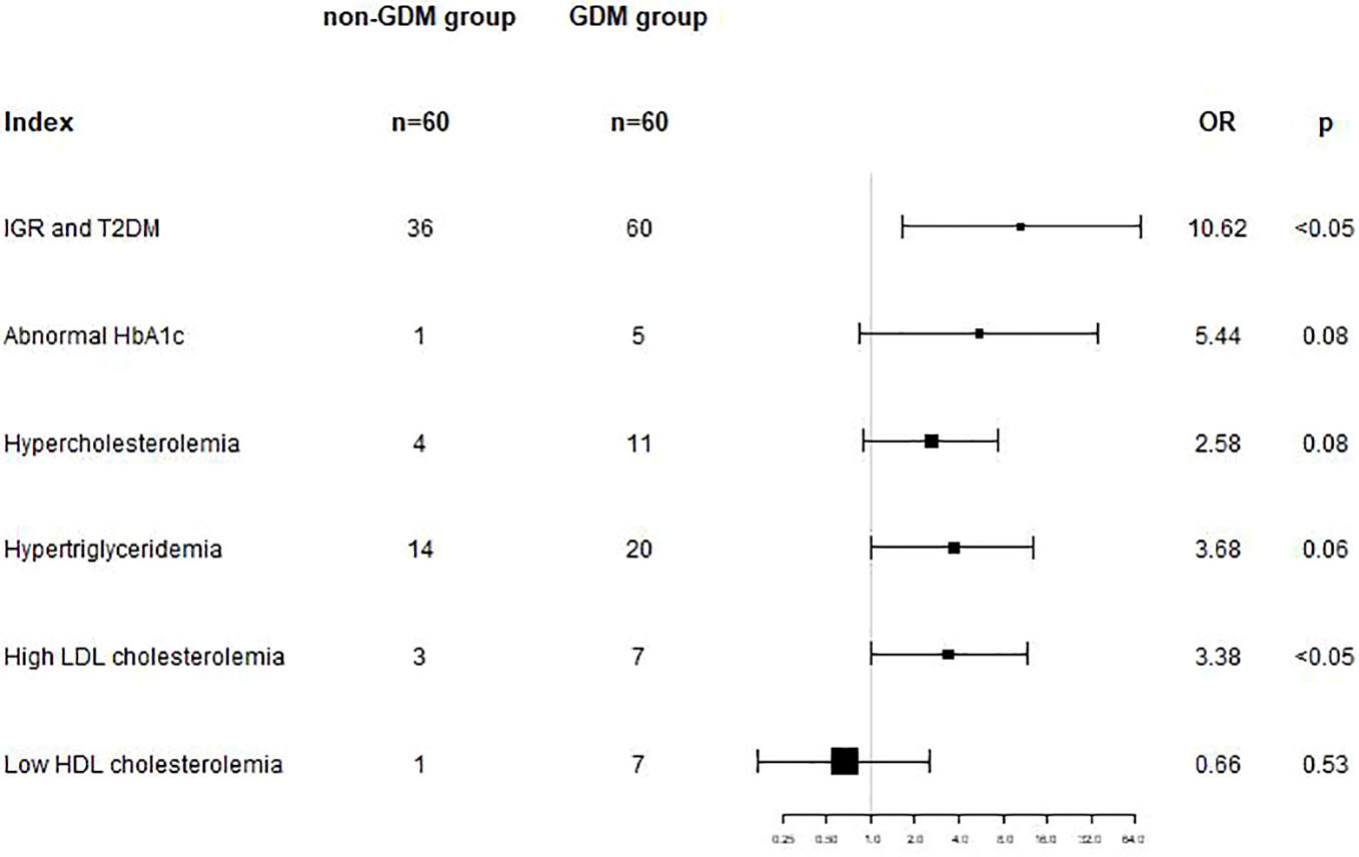
In model 2, adjusted for gestational age, parity and follow-up time, GDM was an independent risk factor for postpartum hyperglycemia (OR=10.62, 95%CI: 1.66-68.17, p<0.05), and for postpartum high LDL cholesterolemia (OR=3.38, 95%CI: 1.01-11.56, p<0.05).

Logistic regression analysis was used to analyze the association between GDM and postpartum metabolic abnormalities. After adjusting for age, parity and follow-up time, the results showed that GDM was an independent risk factor for postpartum hyperglycemia (including T2DM and IGR) (OR=10.62, 95%CI: 1.66-68.17, p<0.05), and GDM was an independent risk factor for postpartum high LDL cholesterolemia (OR=3.38, 95%CI: 1.01-11.56, p<0.05). (shown as **Table 4** and **Figure 1**).

**Table 4:** Correlation between GDM and postpartum metabolic abnormalities.

Index	Mode 1		Model 2	
	OR (95%CI)	p	OR (95%CI)	p
IGR and T2DM	6.51 (1.38,30.79)	<0.05	10.62 (1.66,68.17)	<0.05
Abnormal HbA1c (%)	6.51 (1.38,30.79)	<0.05	5.44 (0.84,35.31)	0.08
Hypercholesterolemia (%)	2.40 (1.03,5.57)	<0.05	2.58 (0.90,7.41)	0.08
Hypertriglyceridemia (%)	1.89 (0.69,5.20)	0.22	3.68 (0.99,12.73)	0.06
High LDL cholesterolemia (%)	2.75 (1.04,7.29)	<0.05	3.38 (1.01,11.56)	<0.05
Low HDL cholesterolemia (%)	0.88 (0.33,2.35)	0.80	0.66 (0.17,2.49)	0.53

Model 1 did not adjust for other factors, Model 2 adjusted for gestational age, parity and follow-up time.

### 3.4 Mediating effect of inflammatory factors on postpartum metabolic indicators in GDM patients

The inflammatory factor IL-6 had a mediating effect in the association between GDM and postpartum GLU_0min_, TG and SDLDL-C, with the mediating effect sizes being 18.18%, 32.43% and 31.25%, respectively; the inflammatory factor IL-6 had a mediating effect in the association between GDM and postpartum TyG and siMS, with the mediating effect sizes being 20.59% and 30.77%, respectively; however, IL-6 did not have a mediating effect in the association between GDM and other postpartum metabolic indicators. Meanwhile, other inflammatory factors such as IL-1β, IL-8, IL-2, TNF-α, TNF-β and GDF15 did not have a mediating effect in the association between GDM and postpartum metabolic indicators (Shown in **Table 5**).

**Table 5:** Mediating effect of IL-6 in the association between GDM and postpartum metabolic indicators.

Index	GDM		IL-6		GDM*IL-6		R^2^	F
	βst (95%CI)	p	βst (95%CI)	p	βst (95%CI)	p		
GLU_0min_	0.63 (0.09,1.18)	<0.05	0.14 (0.02,0.31)	<0.05	0.77 (0.23,1.32)	<0.05	0.11	6.98
HbA1c	0.40 (0.14,0.65)	<0.05	0.04 (-0.02,0.17)	0.11	0.44 (0.19,0.69)	<0.05	0.12	7.61
TyG	0.27 (0.01,0.52)	<0.05	0.07 (0.01,0.20)	<0.05	0.34 (0.09,0.59)	<0.05	0.11	7.37
siMS	0.27 (-0.09,0.63)	0.14	0.12 (0.01,0.32)	<0.05	0.39 (0.03,0.75)	<0.05	0.11	7.18
TC/HDL-C	0.28 (-0.10,0.66)	0.14	0.08 (-0.01,0.19)	0.06	0.36 (-0.01,0.73)	0.06	0.06	3.63
TC	0.42 (0.13,0.72)	<0.05	0.03 (-0.05,0.10)	0.46	0.45 (0.16,0.74)	<0.05	0.08	4.91
TG	0.25 (-0.13,0.65)	0.19	0.12 (0.03,0.23)	<0.05	0.37 (-0.02,0.76)	0.07	0.08	5.05
LDL-C	0.32 (0.06,0.58)	<0.05	0.02 (-0.04,0.09)	0.48	0.34 (0.09,0.59)	<0.05	0.06	3.77
HDL-C	0.03 (-0.08,0.14)	0.57	-0.03 (-0.05,0.00)	0.06	0.01 (-0.10,0.12)	0.86	0.03	1.75
SDLDL-C	0.12 (-0.02,0.26)	0.09	0.05 (0.01,0.08)	<0.05	0.16 (0.02,0.30)	<0.05	0.09	6.00

## 4 Discussion

In recent years, the incidence of GDM in China has increased sharply, becoming an important public health problem. A Meta-analysis of the incidence of GDM in Southeast Asia in 2018 showed that the incidence of GDM in China was 11.91%, much higher than that in Japan, South Korea and Thailand (20). Many studies in the past decade have shown that GDM has adverse effects on postpartum cardiovascular health in women. Compared with women without GDM, women with previous GDM have a two-fold risk of future cardiovascular events. CVD is the leading cause of morbidity and mortality in women worldwide, and also the leading cause of death in Chinese women (21). In the past decades, many studies have explored the association between GDM and postpartum CVD (22). The results of most studies showed that the risk of postpartum CVD in GDM was significantly increased, but the risk factors associated with postpartum CVD risk in GDM have not been clear.

This study comprehensively explored the association between GDM history and postpartum CVD-related metabolic indicators. The results showed that after adjusting for confounding factors, GDM was significantly correlated with postpartum GLU_0min_ (β=0.94, 95%CI: 0.27-1.60, p<0.05), GLU_120min_ (β=2.76, 95%CI: 1.57-3.94, p<0.05), and HbA1c (β=0.49, 95%CI: 0.18-0.79, p<0.05), which was consistent with most previous studies (19–22). Logistic regression analysis showed the association between GDM and postpartum metabolic abnormalities. After adjusting for age, parity and follow-up time, the results showed that GDM was an independent risk factor for abnormal blood glucose (including T2DM and IGR) (OR=10.62, 95%CI: 1.66-68.17, p<0.05). The ways in which abnormal glucose metabolism affects the increased risk of postpartum CVD in GDM can be roughly divided into two categories: one is that GDM progresses to T2DM after delivery, thereby increasing the risk of CVD; the other is that independent of the progression of T2DM, the impact of abnormal glucose metabolism on the postpartum CVD risk in GDM (23). A large population-based retrospective cohort study in South Korea tracked T2DM and CVD events in more than 1.5 million women. The multivariate adjusted results showed that compared with women without GDM, the CVD risk ratio of women with previous GDM was 1.08 (95%CI: 1.02-1.14). Further classification according to the progression of T2DM in GDM showed that the CVD risk of GDM women who had progressed to T2DM was significantly higher (HR=1.74, 95%CI: 1.40-2.15), that is, compared with GDM women who had progressed to T2DM, the CVD risk of GDM women who had progressed toT2DM was significantly higher (24). However, in addition to the CVD risk caused by GDM progression to T2DM, the adverse glucose metabolism of GDM women who did not progress to T2DM also had an independent effect on the increased CVD risk of GDM. In a large-scale postpartum repeated assessment study in Canada, Retnakaran et al. conducted a total of 3 postpartum follow-up visits on 757,541 women at median postpartum 4.8, 7.1 and 8.7 years, respectively, and measured GLU_0min_ and HbA1c to evaluate their blood glucose metabolism, and divided these women into four groups according to the prevalence of GDM and postpartum CVD: GDM−/CVD−, GDM+/CVD−, GDM−/CVD+ and GDM+/CVD+. The results showed that the blood glucose indexes of previously diagnosed GDM women were significantly poorer, and further mediation analysis showed that the main determinants of CVD risk in GDM women were glycosylated hemoglobin (56%) and fasting blood glucose (47.4%), that is, poor postpartum blood glucose metabolism would mediate the increased CVD risk in previously diagnosed GDM women (11).

The specific mechanism of abnormal glucose metabolism leading to CVD occurrence has been discussed in many studies, including (1) Advanced glycation end products(AGE)- receptor of AGE(RAGE) axis, high blood glucose accelerates the formation of AGEs, which accumulate in the extracellular matrix of blood vessels and can induce vascular inflammation and endothelial function injury, promote foam cell formation, down-regulate the number of endothelial cells and accelerate arterial wall sclerosis, etc., leading to CVD occurrence (25, 26). (2) Oxidative stress and nitrogen oxides: diabetes increases vascular oxidative stress and promotes posttranslational oxidative modification of proteins, leading to cell damage and vascular dysfunction. High glucose also induces activation of redox-sensitive protein kinase C, polyols and hexosamine pathways, further leading to mitochondrial dysfunction, endoplasmic reticulum stress and subsequent cell damage. In addition, oxidative stress is also associated with the bioavailability of vasodilator nitric oxide, leading to endothelial dysfunction (27–29). (3) Inflammation and immune system: under abnormal blood glucose levels, monocytes, macrophages and natural killer cells infiltrate into tissues such as fat and muscle, leading to changes in the number and type of immune cells, and the release of proinflammatory factors, leading to chronic inflammation. Inflammatory factors are involved in changes in a variety of signaling pathways, and can affect the occurrence and development of CVD by promoting the proliferation and migration of vascular smooth muscle cells and affecting vascular endothelial function (30, 31).

The results of this study also found the association between GDM and postpartum lipid metabolism indicators. After adjusting age, parity and follow-up time, there was a significant correlation between GDM and postpartum TC (β=0.48, 95%CI: 0.12-0.84, p<0.05), LDL-C (β=0.38, 95%CI: 0.08-0.69, p<0.05), SDLDL-C (β=0.19, 95%CI: 0.02-0.37, p<0.05), APO-b (β=13.59, 95%CI: 3.21-23.97, p<0.05), LPA (β=58.61, 95%CI: 3.97-113.25, p<0.05) and FFA (β=132.00, 95%CI: 29.77-234.23, p<0.05). At the same time, Logistic regression analysis showed that GDM was an independent risk factor for high LDL cholesterolemia (OR=3.38, 95%CI: 1.01-11.56, p<0.05). However, GDM was not associated with other postpartum lipid metabolism indicators, including TG and HDL-C. A study in Hungary found that after adjusting for age and BMI at follow-up, there was a statistically significant difference in triglyceride between GDM (n=68) and control group (n=39) (32). A study in Massachusetts, USA found that after adjusting for age, race, family history of diabetes and other factors, there were differences in glycosylated hemoglobin and triglyceride between GDM group (n=76) and control group (n=461) (33). However, some studies suggested that there was no significant association between GDM history and postpartum lipid metabolism indicators. A large-scale Iranian study with a long postpartum follow-up period showed that after adjusting for age, BMI, and lipid levels at baseline, there was no statistically significant difference in TC, TG, HDL-C, and LDL-C between 289 GDM women (median postpartum follow-up: 7 years) and 1183 controls (median postpartum follow-up: 8 years) (33). A study in Louisiana, USA showed that after adjusting for age, BMI, race, education, income, smoking and drinking, physical activity and dietary intake, there was no statistically significant difference in TC, TG, HDL-C and LDL-C between previous GDM women (n=555) and control group (n=7572) at an average of 22.9 years postpartum. This study followed up for a longer period of time after birth, and after adjusting for confounding factors such as lifestyle, there were no differences in lipid metabolism indexes between the two groups (34). The reasons for the differences in the results of these studies may be due to the sample size, which was small in the study of Hungary (33), and large in the studies of Iran and Louisiana (33, 34). Secondly, there were significant differences in the length of follow-up or the length of postpartum between the GDM group and the control group. The length of postpartum in the GDM group and the control group in the study of Hungary (32) was (3.5 ± 0.6) years and (8.2 ± 5.1) years, respectively, and the difference in the length of postpartum between the two groups was significant (P<0.001), while the length of postpartum in the studies of Iran and Louisiana (33, 34) was more than 7 years. In addition, the study in Hungary (32) adjusted confounding factors and did not consider the impact of other factors on blood lipids. However, many studies have shown that lifestyle factors such as sleep, physical activity, and dietary intake are important influencing factors for lipid metabolism and the risk of CVD (35–37). However, after adjusting many relevant confounding factors, studies in Iran and Louisiana (34, 35) did not find an association between GDM and postpartum lipid metabolism. In addition, differences in the diagnostic criteria for GDM may also be the reason for different results. The GDM diagnostic criteria used in the study in Massachusetts (32) in the United States were two-step methods, while the GDM diagnostic criteria in the Chinese population in this study were one-step methods. Studies have shown that the one-step method has a higher GDM screening rate than the two-step method, and can identify milder GDM patients (38). Therefore, based on the existing research foundation, it is still not possible to determine the specific association between GDM history and postpartum lipid metabolism. In the future, it is necessary to consider exploring the relationship between GDM and postpartum lipid metabolism in different diagnostic criteria with a larger sample size, longer follow-up time, and more comprehensive adjustment of confounding variables.

Previous studies have also shown an association between GDM and postpartum blood pressure level. A prospective study on maternal cardiovascular health in Manchester, UK showed that the systolic and diastolic blood pressures of mothers with previous GDM were significantly higher than those of healthy controls at 2 years postpartum (39). A study in Turin, Italy showed that compared with controls without GDM during pregnancy, the systolic and diastolic blood pressures of GDM women were significantly higher at 6.5 years postpartum, and the systolic and diastolic blood pressures were significantly correlated with carotid intima-media thickness, an indicator of vascular endothelial dysfunction (40), indicating an increased cardiovascular risk of GDM women in the future. This is similar to the results of this study, which showed that after adjusting for age, parity and follow-up time, GDM was correlated with postpartum DBP (β=4.17, 95%CI: 0.17-8.18, p<0.05), and the correlation was statistically significant. However, some studies did not show a correlation between GDM and postpartum blood pressure levels. A cohort study with a follow-up period of 14-16 years and a cross-sectional study in Rio de Janeiro, Brazil both showed that there was no statistically significant difference in postpartum systolic and diastolic blood pressures between previous GDM women and the control group (41, 42). Another case-control study with age as a matching factor also showed no significant difference in systolic and diastolic blood pressures between GDM and the control group at 6 years postpartum (43). In these studies, those that showed an association between GDM and increased postpartum blood pressure tended to have shorter postpartum follow-up, whereas studies with follow-up longer than 6 years did not show such an association.

In recent years, some comprehensive metabolic indicators such as TyG and siMS have also been used to predict the occurrence of CVD. TyG is an index composed of two risk factors for cardiovascular disease, lipid-related and glucose-related factors, which are influencing factors of insulin resistance in human body. Recent studies have determined that TyG is a reliable marker of insulin resistance, which may be one of the explanations for this association (8). A previous study based on NHANES showed that there was a “U”-shaped correlation between baseline TyG and cardiovascular disease in patients with diabetes or prediabetes in the US population, and the thresholds of CVD prevalence and all-cause mortality were 8.84 and 9.05, respectively (44). The siMS score is a simple score that uses waist circumference, height, GLU_0min_, TG, SBP and HDL-C to evaluate metabolic syndrome. Studies have confirmed that the score is significantly correlated with CVD (45). At present, no studies have focused on the correlation between GDM and postpartum TyG and siMS. This study is also the first to explore the correlation between GDM and postpartum comprehensive metabolic indicators. The results showed that after adjusting age, parity and follow-up time, GDM was correlated with postpartum metabolic indicators TyG (β=0.31, 95%CI: 0.01-0.61, p<0.05) and siMS (β=0.45, 95%CI: 0.03-0.88, p<0.05), and the association was statistically significant. This further confirmed that the significant correlation between GDM and postpartum CVD may be caused by metabolic disorders.

This study also conducted the correlation analysis of GDM and postpartum inflammatory factors. The results of linear regression analysis showed that after adjusting age, parity and follow-up time, GDM was significantly correlated with IL-6 (β=0.91, 95%CI: 0.02-1.79, p<0.05), but not with other inflammatory factors. Previous studies have shown that proinflammatory factors can cause insulin resistance by interfering with insulin signaling pathways, such as IKKβ/NF-κB pathway, JNK pathway and inflammasome pathway, and thus increase GLU_0min_ (46). Previous studies have shown that IL-6 in patients with GDM was significantly higher than that in patients without GDM, and may activate intracellular IL-6 signaling and affect the activation of IL-6/IL-6R pathway (47). An Indian study showed that the IL-6 and TNF-α in the third trimester of GDM group (n=35) and control group (n=30) were different (48). Previous studies have confirmed that IL-6 can be used as a diagnostic biomarker for GDM (49). However, there were also studies reporting no significant correlation between GDM and inflammatory factors in the third trimester. A Meta study on the correlation between GDM and inflammatory factors in the second or third trimesters pointed out that the TNF-α in the GDM group was slightly higher than that in the control group, but not significant (50). The existing research on the association between GDM and inflammatory factor levels is still controversial. The main reasons for the difference in research results may be the detection methods of inflammatory factors used in relevant studies are different, such as enzyme-linked immunosorbent assay, chemiluminescence immunoassay and multiple microbead method, etc. The detection methods have different sensitivities, which may affect the ability to detect small differences. Secondly, the sample size of relevant studies is small. The sample size of inflammatory factors in this study is 60 cases in the non-GDM group and 60 cases in the GDM group. The small sample size may also lead to the difficulty in identifying the differences between the two groups.

This study further analyzed the mediating effect of inflammatory factor IL-6 in the association between GDM and postpartum GLU_0min_, TG and SDLDL-C, with the mediating effect sizes being 18.18%, 32.43% and 31.25%, respectively; the mediating effect of inflammatory factor IL-6 in the association between GDM and postpartum TyG and siMS, with the mediating effect sizes being 20.59% and 30.77%, respectively. In a previous systematic review, Google Scholar, Scopus, PubMed, ISI Web of Science, ProQuest, and MEDLINE databases were searched using the following keywords: GDM, screening, and IL-6, with the time interval 2009–2020. The result has shown that the serum IL-6 levels can be investigated a newly established diagnostic biomarker for GDM (49).An recent study showed that the concentrations of IL-6 protein and IL-8 protein in GDM were increased in both maternal and umbilical arterial blood, which suggested that women with GDM exhibit an increased risk of neonatal infection via inflammation and autophagy in the placenta (51). Elevated IL-6 levels have been linked to several adverse cardiovascular outcomes, including an increased risk of myocardial infarction, heart failure, and overall mortality in CVD patients (52). However, no research has yet confirmed that IL-6 is the main factor in postpartum CVD and metabolic diseases in GDM patients. This study demonstrates that IL-6 is not only in the pregnancy period, but also in the correlation between GDM and IL-6, but the abnormal inflammatory factor indicators in GDM patients may continue to postpartum, and become the mediating factor for the increased risk of postpartum CVD in GDM patients. However, the mechanism of IL-6 still needs further animal experiments and cell studies to confirm in GDM.

There were also some limitations in this study. First, the small sample size, this study only included 120 cases of study analysis, may affect the statistical power of the differences found in this study, therefore, the negative results in this study still need to be further verified by other studies in the future. Secondly, this study only detected and analyzed the association between seven inflammatory factors and GDM and postpartum metabolic indicators, and some other important inflammatory indicators, such as IL-4 and some anti-inflammatory factors, were not included in the analysis of this study. In future studies, we will include more comprehensive inflammatory indicators to better reveal the pathophysiological mechanism between GDM and increased risk of postpartum CVD. In addition, our future research needs to focus on the impact of interventions for GDM (medication, personalized therapy, or lifestyle interventions) on the improvement of glucose and lipid metabolism in postpartum women, and further reveal effective methods for managing these diseases.

## 5 Conclusion

This study revealed that the history of GDM will lead to abnormal glucose and lipid metabolism indicators in women after delivery, affect the level of postpartum CVD-related metabolic indicators, and may increase the risk of postpartum CVD. At the same time, IL-6 presented an intermediary role, providing an important clinical basis for the prevention and control of CVD in such high-risk populations and the improvement of cardiovascular health in the population.

## Data Availability

The raw data supporting the conclusions of this article will be made available by the authors, without undue reservation.
